# Synthesis and Characterization of Organic Dyes Containing Various Donors and Acceptors

**DOI:** 10.3390/ijms11010329

**Published:** 2010-01-22

**Authors:** Tzi-Yi Wu, Ming-Hsiu Tsao, Fu-Lin Chen, Shyh-Gang Su, Cheng-Wen Chang, Hong-Paul Wang, Yuan-Chung Lin, Wen-Chung Ou-Yang, I-Wen Sun

**Affiliations:** 1 Department of Chemistry, National Cheng Kung University, Tainan, Taiwan; 2 Sustainable Environment Research Center, National Cheng Kung University, Tainan 701, Taiwan; 3 Department of Environmental Engineering, National Cheng Kung University, Tainan, Taiwan; 4 Institute of Environmental Engineering, National Sun Yat-Sen University, Kaohsiung 804, Taiwan; 5 Department of Chemical and Materials Engineering, National Kaohsiung University of Applied Sciences, Kaohsiung 80778, Taiwan

**Keywords:** organic dyes, donor, absorption, electrochemistry, photovoltaic materials

## Abstract

New organic dyes comprising carbazole, iminodibenzyl, or phenothiazine moieties, respectively, as the electron donors, and cyanoacetic acid or acrylic acid moieties as the electron acceptors/anchoring groups were synthesized and characterized. The influence of heteroatoms on carbazole, iminodibenzyl and phenothiazine donors, and cyano-substitution on the acid acceptor is evidenced by spectral, electrochemical, photovoltaic experiments, and density functional theory calculations. The phenothiazine dyes show solar-energy-to-electricity conversion efficiency (*η*) of 3.46–5.53%, whereas carbazole and iminodibenzyl dyes show *η* of 2.43% and 3.49%, respectively.

## 1. Introduction

Donor–acceptor (D–A) organic molecules are among the most important conjugated organic materials, and have attracted much academic and technological research interest [1]. In these compounds the electron-donating and electron-accepting groups are connected through a π-conjugated linker. Tuning different donor moiety or acceptor moiety in a D–A molecule would modify its physical and chemical properties. The molecules with D–π–A structures have attracted increasing attention since they can serve as electroactive and photoactive materials in molecular electronics, such as biochemical fluorescent technology [2], efficient nonlinear optical (NLO) materials [3], electrogenerated chemiluminescence [4], organic light-emitting diodes (OLEDs) [5], and solar cells [6].

So far, many organic donor-π-acceptor (D-π-A) compounds have been studied experimentally and theoretically. Numerous species, including triarylamines [7], carbazoles [8], fluorenes [9], thiophenes, and oligothiophenes [10] have mostly been used as electron-donating moieties, whereas oxadiazoles [11], diarylborons [7], quinolines [12], quinoxalines [13], thienopyrazines [14], and benzothiadiazoles [15] are commonly used as electron-accepting moieties. In these compounds, the donor moiety facilitates hole injection and transport, whereas the acceptor moiety facilitates electron injection and transport [16]. Carbazole, *N,N*-dimethylbenzenamine, and phenothiazine are often adopted as donors as a result of their good thermal, electrochemical stability, and donating abilities [17]. The UV–vis absorption and photoluminescence (PL) of these compounds suggest significant intramolecular charge transfer (ICT) behavior and solvatochromism. The electrochemistry shows that the energy level of these compounds can be changed by altering the different donor moieties. Their photovoltaic properties were studied previously [6]. Recently, phenothiazine and its derivatives, outstanding heterocyclic compounds with high electron-donor ability, are attracting more research interest because of their potential applications in materials science, phenothiazines are also active in pharmacology as effective pharmacophores in tranquilizers, antituberculosis agents, antitumor agents, and bactericides [18].

Dye sensitizers, which function as light absorbers, are mainly divided into two kinds: one is metal complexes (ruthenium) and the other is metal-free organic dyes. Recently, more and more attention has been directed to the application of metal-free organic dyes in DSSCs because organic dyes do not require expensive ruthenium which is a limited resource, their relatively facile dye synthesis, and easy molecular tailoring. Pure organic dyes usually have much stronger light-harvesting ability than metal complexes because of their high extinction coefficients and very rich photophysical properties, it is promising to improve photocurrent to the theoretical maximum through molecular design of metal-free organic dyes. The organic dyes commonly consist of donor, linker, and acceptor groups (*i.e.*, a D-π-A molecular structure). Their properties can be finely tuned by independently alternating or matching the different groups of D-π-A dyes. In the research for organic solar cells with high efficiency, the development of new materials offering optimized thermal and photochemical stabilities, optical and electrical properties. However, π-π stacking of organic dye molecules usually occurs because of the strong intermolecular interaction. Although π-πstacking is advantageous to light harvesting because of its broad feature in UV-vis absorption spectrum, π-stacked aggregate usually leads to inefficient electron injection and thus results in low power conversion efficiency [19]. Prohibition of π-π stacking with additive in the dye solution is a typical way to improve efficiency of organic dye-sensitized solar cells suffering from the π-π stacking problem. Coadsorption of dye with additives [20] and structural modification of dye molecules [21] are proven to be effective to dissociate π-π stacking or dye aggregation and thus to improve solar cell efficiency.

In this work, new metal-free dyes with carbazole, iminodibenzyl, and phenothiazine sensitizers, respectively, are reported and their optical, electrochemical, photovoltaic properties, and structural relationships are investigated in detail. We synthesized four donor-acceptor π-conjugated dyes with a carboxyl group; the amine derivatives act as the electron donors while a 2-cyanoacrylic acid (or acrylic acid) moiety acts as the anchoring group for attachment on the metal oxide and as the electron acceptor. The two parts are connected by a π-conjugated methane unit. We investigate the electron-donating and electron-accepting nature, structural variations of the amine and acid unit, respectively, and study their electronic properties.

## 2. Results and Discussion

### 2.1. Synthesis and Structure of Sensitizers

**Figure 1.**
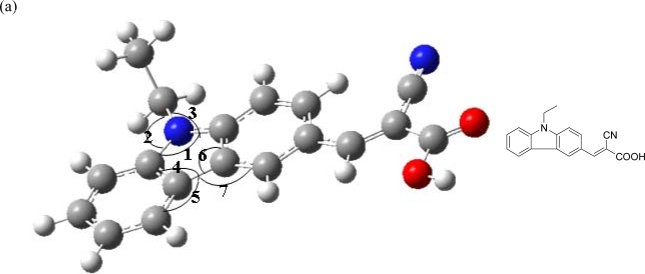

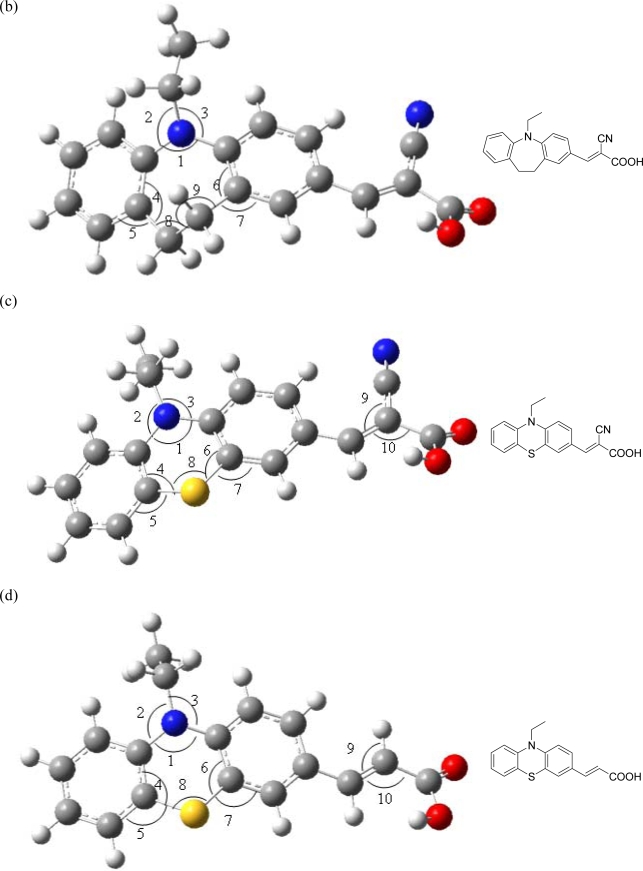
Optimized geometric parameters (dihedral angle) of (a) **D1**: ∠1 = 108.7°, ∠2 = 125.4°, ∠3 = 125.9°, ∠4 = 106.4°, ∠5 = 134.0°, ∠6 =106.6°, ∠7 =134.0°; (b) **D2**: ∠1 = 123.7°, ∠2 = 118.9°, ∠3 = 117.3°, ∠4 = 126.1°, ∠5 = 115.7°, ∠6 = 119.4°, ∠7 = 121.5°, ∠ 8 = 116.2°, ∠9 = 111.1°; (c) **D3**: ∠1 = 122.1°, ∠2 = 118.3°, ∠3 = 118.8°, ∠4 = 120.6°, ∠ 5 = 118.3°, ∠6 = 120.4°, ∠7 = 118.5°, ∠8 = 99.2°, ∠9 = 123.8°, ∠10 = 121.8°; (d) **D4**: ∠1 = 121.8°, ∠2 = 118.5°, ∠3 = 118.7°, ∠4 = 120.5°, ∠5 = 118.3°, ∠6 = 120.4°, ∠7 = 118.4°, ∠8 = 99.2°, ∠9 = 121.6°, ∠10 = 125.1°.

As shown in Figure 1, **D1**–**D3** are cyanoacetic acid-based dyes with carbazole, iminodibenzyl, and phenothiazine unit as the donor, respectively. **D4** is an acrylic acid-based dye with a phenothiazine unit. The aldehydes **3**, **6**, **9** were prepared by a Vilsmeier reaction of *N*-ethylcarbazole, *N*-ethyliminodibenzyl, and *N*-ethylphenothiazine, respectively, with POCl_3_ in DMF [22]. The final reaction for **D1**–**D4** was the condensation of the respective aldehyde with cyanoacetic acid (or malonic acid) by the Knoevenagel reaction in the presence of piperidine. The optimized structures of **D1**–**D4** were studied to compare the structure differences between these photosensitizers (Figure 1). The dihedral angles (∠1) of the **D1** carbazole ring, the **D2** iminodibenzyl ring, and the **D3** phenothiazine ring are 108.7°, 123.7°, and 122.1°, respectively, implying that the addition of a dimethylene bridge and sulfur units significantly increases the dihedral angle of nitrogen atoms. The dihedral angle between two phenyl units inside the carbazole unit (∠4) of **D1** is 106.4°; however, the dihedral angle between the phenyl unit and dimethylene bridge inside the iminodibenzyl unit (∠4) of **D2** is 126.1°. This can be attributed to the incorporation of a dimethylene bridge between two phenyl units increasing the steric hindrance, increasing the dihedral angles of ∠1 and ∠4. The dihedral angle (∠8) of the **D3** phenothiazine sulfur atom is 99.2°, which is smaller than that of the phenothiazine nitrogen atom (∠1: 122.1°). This may be attributed to the difference between nitrogen and sulfur atoms; nitrogen atom has a lone pair whereas sulfur atom has two lone pairs. For the cyanoacetic acid and acrylic acid-based dye, the dihedral angles (∠9) of the **D3** and **D4** are 123.8° and 121.6°, respectively, whereas dihedral angles (∠10) of the **D3** and **D4** are 121.8° and 125.1°, respectively implying that the incorporation of a electron-withdrawing cyano group decreases the dihedral angle (∠10).

### 2.2. Optical Properties

**Figure 2.**
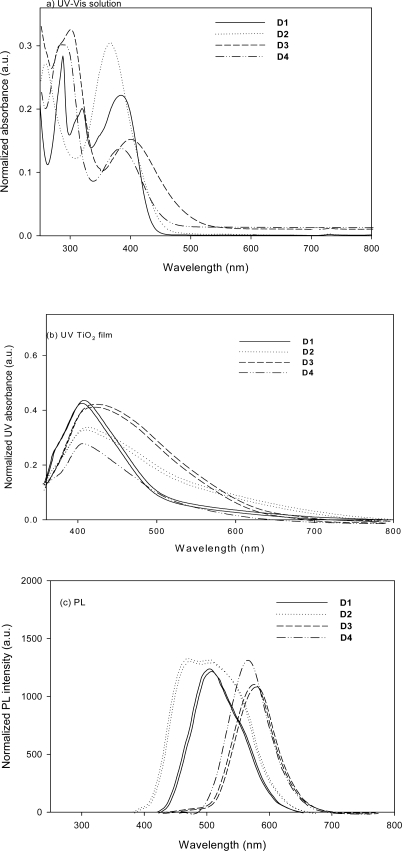
Absorption and emission spectra of **D1**–**D4** in CH_3_CN and absorption spectra of **D1**–**D4** adsorbed on TiO_2_ film.

Figure 2 shows the UV-Vis absorption and emission spectra for the four dyes in CH_3_CN and the λ_max_ are listed in Table 1, together with the UV–Vis spectra of the corresponding dyes adsorbed on TiO_2_ film. The absorption spectrum of **D1** in CH_3_CN has three distinct absorption bands at around 288, 320 and 385 nm, respectively. The absorption peaks at around 320 nm correspond to the π→π* electron transition of the conjugated molecule; and the absorption peaks at around 385 nm can be assigned to an intramolecular charge transfer between the carbazole-based donor and the cyanoacetic acid [23], providing efficient charge-separation at the excited state. Under similar conditions, the **D2** sensitizer had absorption peaks at 258 and 368 nm that were blue-shifted relative to the peaks of **D1**, implying that the incorporation of dimethylene bridge destroys the conjugation between two phenyl groups. Compare the maximum absorption wavelength of **D1**–**D3** in CH_3_CN solution. **D3** (400 nm) shows a red shift relative to **D1** (385 nm) and **D2** (368 nm), which can be attributed to the extra electron-donating sulfur atom in **D3**. Such red-shifting in the absorption spectra implies a more effective utilizing of solar light. For the cyanoacetic acid and acrylic acid-based dyes (**D3** and **D4**, respectively), when an electron acceptor (-CN) was linked to the vinyl bridge, the maximum absorption was red-shifted from 381 to 400 nm. The red shift of the maximum absorption peak arises from the fact that one more electron acceptor (-CN) enhances the electron-withdrawing ability of electron acceptors and lowers the LUMO, thus reducing the gap between HOMO and LUMO.

Figure 2(b) shows the UV-Vis absorption spectra for the dye loaded TiO_2_ films with a bare TiO_2_ film as the reference. Upon dye adsorption onto the TiO_2_ surface, the maximum absorption respectively red-shifted by 23, 46, 26, and 31 nm for **D1**, **D2**, **D3**, and **D4** as compared to the spectra in solution, implying that dyes adsorbed on the TiO_2_ surface had partial J-type aggregates. However, the stronger red shift of **D2** (368 nm→414 nm) compared to that of **D1** (385→408 nm) from the solution to TiO_2_ film may imply that the iminodibenzyl-containing sensitizer has a higher tendency to form surface binding with TiO_2_ than that of carbazole-containing sensitizer, as chelating to metal cation, it may enhance the intramolecular electron transfer (ICT), leading to spectrum shift. Under similar conditions, the **D3** and **D4** sensitizers showed a slight red shift (400→426 nm and 381→412 nm, respectivity) after being adsorbed on the TiO_2_ surface.

The absorption spectra of **D1**–**D4** on the TiO_2_ electrode are broader than those in CH_3_CN. The broadening of the absorption spectra is due to an interaction between the dyes and TiO_2_ [24]. The absorption redshift of **D3** relative to **D2** is due to its stronger intramolecular charge transfer because phenothiazine is a stronger electron-donating ring than iminodibenzyl. The molar extinction coefficients of **D1**, **D2**, **D3**, and **D4** in CH_3_CN solution are 22,975 M^−1^ cm^−1^ (at 385 nm), 29,846 M^−1^ cm^−1^ (at 368 nm), 14,096 M^−1^ cm^−1^ (at 400 nm), and 12,639 M^−1^ cm^−1^ (at 381 nm), respectively; they are comparable or larger than that of **N3** (*cis*-bis(isothiocyanato)bis(2,2′-bipyridyl-4,4′-dicarboxylato)-ruthenium(II)) (14,200 M^−1^ cm^−1^) [25], indicating that the novel dyes have good light harvesting ability.

As shown in Figure 2 (c), the PL maxima of **D1** are located at 504 nm in CH_3_CN, whereas those of **D2** shifted to 470 and 506 nm, and those of **D3** and **D4** shifted to 573 and 558 nm, respectively. **D3** and **D4** exhibited a relatively large Stokes shift (173 and 177 nm, respectively), which could be attributed to the geometrically relaxed structure of the phenothiazine center upon excitation [26].

### 2.3. Electrochemical Properties

**Figure 3.**
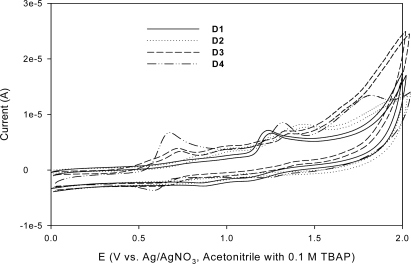
Cyclic voltamogram of **D1**–**D4** in acetonitrile.

To judge the possibilities of electron transfer from the excited dye molecule to the conductive band of TiO_2_ and the dye regeneration, redox potentials of **D1**–**D4** in acetonitrile were obtained using cyclic voltammetry (as shown in Figure 3). The electrochemical data are listed in Table 2. The excited-state oxidation potentials were obtained from the first oxidation potential E_ox_ (*vs.* NHE) measured by cyclic voltammetry, the ferrocenium/ferrocene (Fc/Fc^+^) redox couple was used as an internal potential reference. The excited-state oxidation potential *E*(S^+^/S*) was calculated using Equation 1 [27].

(1) $$E({\text{S}}^{+}/\text{S}*)=E({\text{S}}^{+}/\text{S})-E_{0-0}$$

where *E*(S^+^/S) is the ground state oxidation potential of **D1**–**D4** and *E*_0–0_ is the zeroth–zeroth transition value obtained by the intersection of the normalized lowest energy absorption peak and highest energy fluorescence peak. All *E*_0–0_ values in our experiment were obtained by the absorption and fluorescence spectra measured in acetonitrile which was also used in the preparation of sandwich cell.

When **D1** was swept in the anodic direction, there was an oxidation peak at 1.25 V, which corresponded to the first oxidation steps (formation of the radical cation). However, there was no reduction peak, indicating that the electrochemical behavior in the anodic direction was irreversible. For **D2**, the oxidation was also irreversible, with peaks appearing at 1.00 and 1.42 V, which corresponded to the first and second oxidation steps (forming the radical cation and dication), respectively. They are higher than those of **D1**, which could be attributed to the relatively higher electron density on the nitrogen atoms of **D2**, which allowed it to be more easily oxidized than a carbazole derivative.

It is evident that the iminodibenzyl unit enhanced the hole transporting ability of the photosensitizer more than that of the carbazole unit. The oxidation of **D3** was quasi-reversible, with peaks located at 0.72 and 1.35 V. Both the first and second oxidation potentials are smaller than those of **D2,** indicating that the phenothiazine units are much more effective in lowering the ionization potential than iminodibenzyl units. The oxidation of **D4** was also quasi-reversible, with three peaks located at 0.63, 1.29, and 1.76 V, comparison of these results with previously reported redox properties of phenothiazine-containing oligomers [28] allow assignment of the three waves to the phenothiazine radical cation, dication, and trication, respectively. The irreversibility of the second and third oxidation waves likely stems from the highly reactive nature of the dication and trication [28]. Both the first and second oxidation potentials of **D3** (0.72 and 1.35 V) are larger than those of **D4** (0.63 and 1.29 V), indicating that the incorporation of electron-withdrawing cyano groups at the vinyl bridge enhanced electron affinity significantly. Moreover, introducing a phenothiazine group into the photosensitizer framework (**D3** and **D4**) resulted in a largely negative shift of the HOMO level, to 0.52–0.54 V *vs.* NHE. The HOMO levels of **D1** and **D2** (1.14 and 0.77 V *vs.* NHE, respectively) are more positive than that of **D3**, indicating more efficient dye regeneration for **D1** and **D2**. For the cyanoacetic acid and acrylic acid-based dyes (**D3** and **D4**, respectively), the incorporation of electron-withdrawing cyano groups at the vinyl bridge increases the LUMO level [−2.04 V (**D4**)→−1.92 V (**D3**)]. The LUMO level of **D2** (−2.14 V *vs.* NHE) is more negative than those of **D1**, **D3**, and **D4** (−1.70, −1.92 V, and −2.04 *vs.* NHE, respectively), indicating that **D2** dye has more efficient electron injection.

**Figure 4.**
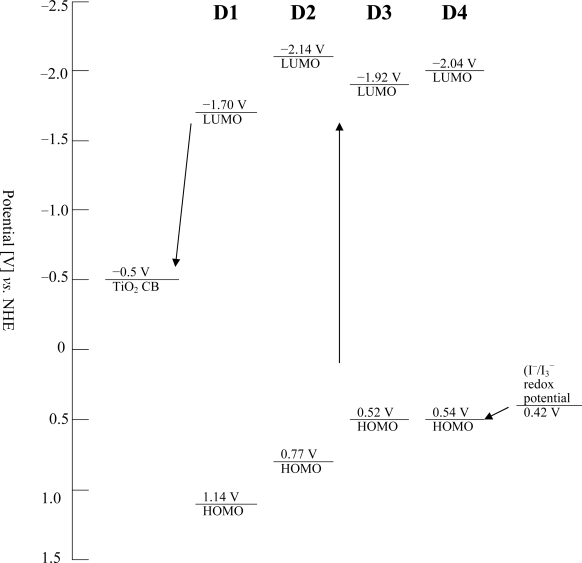
Schematic energy level diagram for a DSSC based on dyes.

Figure 4 shows the schematic energy diagram of these dye-sensitized TiO_2_ electrodes. All the excited state oxidation potentials (LUMO) of **D1** (−1.70 V), **D2** (−2.14 V), **D3** (−1.92 V), and **D4** (−2.04 V) are more negative than the conduction band edge of TiO_2_ [−0.5 V (*vs.* NHE)] [29]. Provided that an energy gap (between dye LUMO and TiO_2_ CB) of 0.2 eV is necessary for efficient electron injection [30], the driving force is sufficient for efficient charge injection. Thus, the electron injection process from the excited dye molecule to the TiO_2_ conduction band and the subsequent dye regeneration are energetically permitted. Figure 4 also indicates that the energy levels of the ground state (HOMO) of **D1** (1.14 V), **D2** (0.77 V), **D3** (0.52 V), and **D4** (0.54 V) are sufficiently more positive than the I_3_^−^/I^−^ redox potential (0.42 V (*vs.* NHE)) [29], indicating that the oxidized dye formed after electron injection into the conduction band of TiO_2_ could accept electrons from I^−^ ions in the electrolyte thermodynamically. Such electronic structures thus ensure a favorable exothermic flow of charges throughout the photo-electronic conversion.

### 2.4. Molecular Orbital Calculations

**Figure 5.**
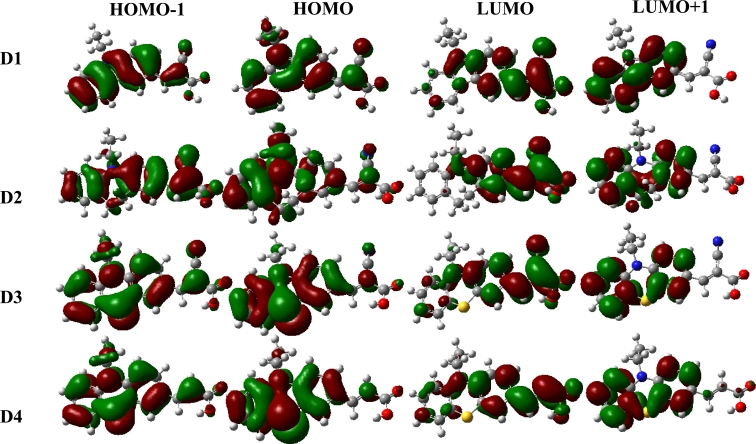
Computed isodensity surfaces of HOMO and LUMO orbitals of **D1–D4**.

The geometrical and electronic properties of **D1**–**D4** were studied with density functional theory (DFT) using the Gaussian 03 program package [31], as shown in Figure 5. The calculations were done on a B3LYP/6-31G(d) level for geometry optimizations in the ground state. B3LYP is a hybrid function modified from the three-parameter exchange-correlation functional of Becke [32], while the gradient-corrected exchange and correlation functions are calculated according to Becke [33] and Lee *et al*. [34]. The frontier MOs of **D2**–**D4** reveal that HOMO–LUMO excitation moves the electron density distribution from the iminodibenzyl, phenothiazine moiety to the cyanoacrylic acid (or acrylic acid) moiety; however, the frontier MOs of **D1** reveal that the electrons at the ground state (HOMO) are homogeneously distributed in both the electron donor group and the methylene bridge of the cyanoacrylic acid. Comparing the frontier MOs of **D2** and **D3** (or **D4**) at the ground state (HOMO), the electrons of **D2** are dominantly distributed in the benzene ring and the nitrogen atom; however, there are few electrons in the dimethylene bridge of the iminodibenzyl unit, the electrons of **D3** (or **D4**) are homogeneously distributed in the phenothiazine unit. The LUMO electron density geometry distributions of **D1–D4** are located over the cyanoacrylic (or acrylic) group through the right phenyl group and the nitrogen atom of carbazole, iminodibenzyl and phenothiazine units, respectively. However, the LUMO + 1 of **D1–D4** is centered on the phenyl group of carbazole, iminodibenzyl, and phenothiazine units. At the excited state (LUMO) with light illumination for **D1–D4** dyes, intramolecular charge transfer occurs, resulting in electron movement from the donor groups to the acceptor groups (cyanoacrylic and acrylic groups). Furthermore, the location of the LUMO at the side of the TiO_2_ surface and the HOMO at the opposite end of the molecule makes the electron injection into the semiconductor easier and prevents back regeneration of the dye with injected electrons.

The vertical excitations were calculated by time dependent density functional theory (TDDFT) with B3LYP/6-31 + G(d) *in vacuo* and in CH_3_CN solution. It was found that the inclusion of solvation effects does not lead to a qualitative change in the electronic structure, even though smaller HOMO-LUMO gaps were calculated in CH_3_CN as compared to the gas phase. The origins of these electronic absorptions were found by calculating the singlet electronic transitions with the TDDFT method in Gaussian 03W program suite. Calculations show that these visible bands are mainly attributed to the electronic transition from the highest occupied molecular orbitals (HOMOs) to the lowest unoccupied molecular orbitals (LUMOs). Table 3 shows the calculated absorption maxima in the visible region, their oscillator strengths, and their compositions for **D1**–**D4**. To analyze the photophysical properties of these dyes, we performed TD-DFT calculations of the lowest 10 singlet–singlet excitations of dyes in CH_3_CN solutions. The three transitions of **D1**–**D4** with oscillator strengths (*f*) above 0.05 are summarized in Table 3. Compared with the experimental data, the considerable blue-shift of the absorption maximum from the calculations is related to the self-interaction error in TD-DFT arising from the electron transfer in the extended charge-transfer state [35]. The calculated absorption maxima for the excited states seem in good agreement with the measured values, supporting the band assignments in the UV solution plot.

### 2.5. Photovoltaic Performance

DSSCs were prepared and compared to investigate the relationships between the sensitizing behavior of **D1–D4** dye molecules and their structures. The dye-sensitized solar cells were constructed by using these dyes as a sensitizer for nanocrystalline anatase TiO_2_.

The incident photon-to-current conversion efficiency (IPCE) of a DSSC was calculated by normalizing the photocurrent densities for incident light energy and intensity according to the Equation 2 [36]:

(2) $$\text{IPCE}(\lambda )=\frac{1240(\text{eV}\cdot \text{nm})}{\lambda (\text{nm})}\frac{J_{sc}(\text{mA}\cdot {\text{cm}}^{-2})}{\varphi (\text{mW}\cdot {\text{cm}}^{-2})}$$

where *J*_sc_ is the short-circuit photocurrent generated by monochromatic light, *λ* is the wavelength of incident monochromatic light, and φ is the power of the incident radiation per unit area.

**Figure 6.**
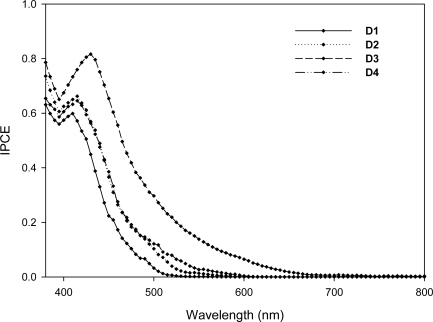
The incident photon-to-current conversion efficiency spectra for DSSCs based on **D1**–**D4**.

Figure 6 shows action spectra of monochromatic incident-to current conversion efficiencies (IPCEs) for DSSCs using **D1**–**D4**. The IPCEs of **D1–D4** are more than 50% in the spectral range from 400 to 430 nm. **D3** reaches its maximum of 82% at 430 nm, whereas **D1**, **D2**, and **D4** reach their maximums of 60–66% at 410–420 nm. When reflection and absorption losses of the FTO glass substrate are considered, the net photon-to-electron conversion efficiencies of the **D1**–**D4** would almost exceed 85% in their spectral ranges. The decrease of the IPCE above 600 nm in the long-wavelength region is attributed to the decrease of light harvesting for these dyes. The lower photocurrent response of **D1**, **D2**, and **D4** sensitized devices is ascribed to the blue shifted dyes with a higher energy band-gap compared to that of the **D3** dye.

**Figure 7.**
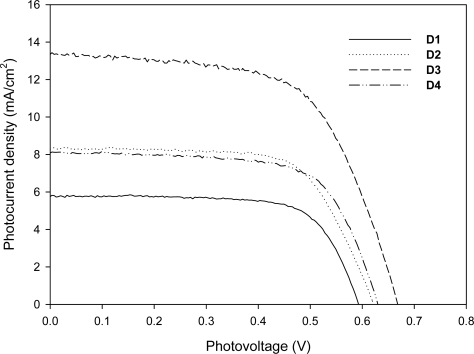
Current density-voltage characteristics for **D1**–**D4** DSSCs under illumination of simulated solar light (AM 1.5, 100 mW·cm^−2^).

A typical photocurrent–photovoltage (*I – V*) curve for cells based on **D1**–**D4** is shown in Figure 7. The detailed photovoltaic parameters are summarized in Table 4. The solar-energy-to-electricity conversion efficiency (*η*) of the DSSCs is calculated from short-circuit current (*J*_sc_), the open-circuit photovoltage (*V*_oc_), the fill factor (FF), and the intensity of the incident light (*P*_in_) according to the following Equation [36]:

(3) $$\eta =\frac{\left[J_{sc}(\text{mA}\cdot {\text{cm}}^{-2})][V_{oc}(\text{V})][\text{FF}\right]}{P_{in}(\text{mW}\cdot {\text{cm}}^{-2})}$$

Under the standard global AM 1.5 solar condition, the **D1** cell had a short circuit photocurrent density (*J*_sc_) of 5.76 mA·cm^−2^, an open-circuit voltage (*V*_oc_) of 0.595 V, and a fill factor of 0.71, corresponding to an overall conversion efficiency of 2.43%. Under similar conditions, the photovoltaic parameters (*J*_sc_, *V*_oc_, and *η*) of cells with the **D2** sensitizer are 8.28 mA·cm^−2^, 0.62 V, and 3.49%, respectively, those of the **D3** sensitizer are 13.35 mA·cm^−2^, 0.669 V, and 5.53%, respectively, and those of the **D4** sensitizer are 8.09 mA·cm^−2^, 0.628 V, and 3.46%, respectively. The great efficiency of the **D3** sensitizer is probably due to the strong electron-donating ability of the phenothiazine unit when electrons transfer from the phenothiazine unit to the cyanoacrylic group. The solar-energy-to-electricity conversion efficiency of the present organic dyes could be improved by extending the conjugated length of organic dyes or incorporating a thiophene π-bridge. The synthesis of high efficiency organic dyes in our lab is still in progress. For the stability or long-term durability of presented organic dyes, the efficiencies of devices don’t show obvious decay till 400 hr, the main contribution of DSSC efficiency decay after 400 hr is the electrolyte. Furthermore, the larger V_oc_ of **D3** is attributed to the retardation of charge recombination at the nanocrystallite/dye/redox electrolyte interface [37], the nonlinear *I* – *V* curve observed in Figure 7 is intriguing, the schematic energy level diagram (Figure 4) for a DSSC based on **D3** shows less energy gap between HOMO *vs.* (I^−^/I_3_^−^) redox potential than those of **D1** and **D2**, this may result in slow diffusion current of main carrier and nonlinearity in *I* – *V* curve.

### 2.6. The Transformation of Solar Energy into Electricity in Photovoltaic Devices

**Figure 8.**
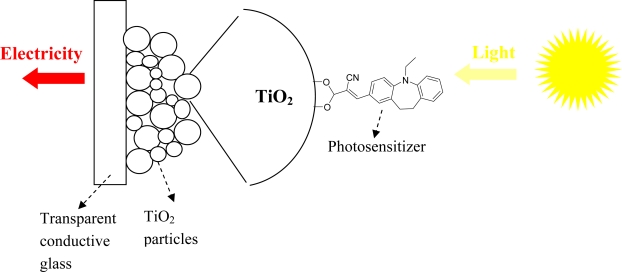
Photovoltaic devices for the transformation of solar energy into electricity.

The conversion of solar energy into electricity is attracting more attention as non-renewable energy reserves diminish. Dye sensitised solar cells consist of a nanostructured semiconductor film (e.g., ~10 μm *anatase titania*, Figure 8), coated with a monolayer of photoactive dye and an electrolyte bearing a redox couple (e.g., I_3_^−^/I^−^). Sunlight enters the cell through the transparent glass plate striking the dye on the surface of the TiO_2_. Absorption of a photon of light by the dye promotes an electron is transferred from S° to a higher lying energy level, then the sensitizer is in the excited state S*. The excited electron is injected into the conduction band of the semiconductor. A carboxyl group in **D1**–**D4** can form an ester linkage with TiO_2_ surface to provide a strongly bound dye and a good electron communication between them.

**Figure 9.**
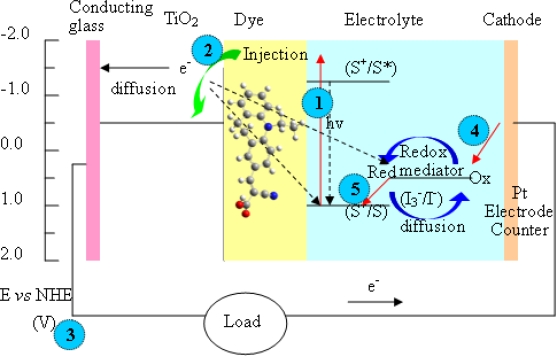
Energy diagram mechanism for a DSSC based on the **D2** photosensitizer, I^−^ /I_3_^−^ redox electrolyte, TiO_2_ anode, and Pt cathode.

The general operating principle of a dye-sensitized solar cell comprising a photoanode (TiO_2_) and a passive cathode is depicted in Figure 9. The regenerative process in the dye solar cell consists of the following five steps, the process leads to photovoltaic performance in the solar cell.

1. Electrons in dye are excited by solar energy adsorption.
2. Electrons are transferred from dye to Indium tin oxide (ITO) conducting glass via TiO_2_.
3. Electrons get to counter electrode after working at external load.
4. I_3_^−^ + 2e^−^→3I^−^ at counter electrode.
5. 3I^−^→I_3_^−^ + 2e^−^ at dye.

## 3. Experimental Section

### 3.1. Chemicals

All starting materials were purchased from Aldrich, Lancaster, TCI, and Acros and used as received. DMF, dichloromethane, and POCl_3_ were distilled from CaH_2_ under N_2_ atmosphere. Tetrabutylammonium perchlorate (TBAP) and 1,2-dimethyl-3-propylimidazolium iodide (DMPImI) were synthesized and purified according to a procedure described in the literature [38].

### 3.2. Instrumentation

The ^1^H-NMR spectra were obtained on a Bruker 400 MHz FT-NMR. Chemical shifts are reported in ppm relative to tetramethylsilane δ units. The absorption spectra of the dyes in solution and adsorbed on TiO_2_ films were recorded on a Cary 100 UV-vis spectrophotometer. Fluorescence measurements were carried out using a Hitachi F-4500 fluorescence spectrophotometer. Cyclic voltammetry was performed using an electrochemical workstation (CH instruments Inc., CHI, model 750A) and conducted in CH_3_CN solution using 0.1 M tetrabutylammonium perchlorate (TBAP) as the supporting electrolyte. The working electrode was a glassy carbon electrode, the auxillary electrode was a Pt wire, and the reference electrode was Ag/Ag^+^. The scan rate was 100 mv·s^−1^ and the temperature was 25 °C. Ferrocene was added to each sample solution at the end of the experiments. The ferrocenium/ferrocene (Fc/Fc^+^) redox couple was used as an internal potential reference. The potential versus SCE in acetonitrile was calibrated according to a procedure published by Matsui *et al*. [39].

### 3.3. Synthetic Procedure of Carbazole, Iminodibenzyl, and Phenothiazine-containing Dyes **D1–D4** (Figure 10)

#### 3.3.1. 11-Ethyliminodibenzyl (**5**)

Iminodibenzyl (**4**: 6.834 g, 35 mmol), iodoethane (5.93 g, 38 mmol), and DMF (80 mL) were added to a 50-mL two-necked glass reactor. The solution was warmed to 75 °C and treated portionwise with potassium *tert*-butoxide (4.264 g, 38 mmol), and then refluxed for 24 h. After water (150 mL) was added, the mixture was extracted with chloroform (75 mL). Crude oils obtained by removing the solvent were purified by column chromatography (silica gels, *n*-hexane/ethyl acetate: 20:1 as eluent) to give **5** as a white solid (4.76 g, 61%). ^1^H-NMR (CDCl_3_), δ (ppm): 1.16 (t, 3H, CH_3_), 3.17 (m, 4H, CH_2_), 3.80 (m, 2H, CH_2_), 6.92 (m, 2H, ar), 7.12 (m, 6H, ar); Elem. Anal. Calcd. for C_16_H_17_N: C, 86.05%; H, 7.67%; N, 6.27%. Found: C, 85.96%; H, 7.73%; N, 6.20%.

#### 3.3.2. 11-Ethyl-3-formyliminodibenzyl (**6**)

POCl_3_ (7.4 g, 48 mol) was added dropwise to freshly distilled *N*,*N*-dimethylformamide (4.3 g, 58 mmol) at 0 °C under a nitrogen atmosphere. A solution of 11-ethyl-iminodibenzyl (**5**: 3.35 g, 15 mmol) in *N,N*-dimethylformamide (4 mL) was added dropwise to the POCl_3_/DMF complex at 30 °C. The reaction mixture was stirred at 80 °C for 4 h. When the reaction was finished (TLC monitoring), the reaction mixture was cooled to room temperature and poured into ice water. The obtained mixture was neutralized with NaOH until pH = 7–8. The precipitates were separated by filtration and washed with methanol. The crude product was purified by column chromatography (eluent: ethylacetate–hexane, 1:6). Yield of **6**: 3.2 g (85%); ^1^H-NMR (CDCl_3_), δ (ppm): 1.07 (t, 3H, CH_3_), 3.11 (m, 4H, CH_2_), 3.85 (m, 2H, CH_2_), 7.00–7.08 (m, 1H, ar), 7.19–7.28 (m, 4H, ar), 7.57 (s, 1H, ar), 7.64–7.67 (m, 1H, ar), 9.78 (s, 1H, CHO); Elem. Anal. Calcd. for C_17_H_17_NO: C, 81.24%; H, 6.82%; N, 5.57%. Found: C, 81.04%; H, 6.90%; N, 5.46%.

#### 3.3.3. 2-Cyano-3-(11-ethyliminodibenzyl-2-yl)-acrylic acid (**D2**)

A mixture of **6** (1.005 g, 4 mmol) and cyanoacetic acid (0.68 g, 8 mmol) was vacuum-dried. Acetonitrile (20 mL) and piperidine (0.341 g, 4 mmol) were added to the mixture. The solution was refluxed for 6 h. After the solution was cooled, the organic layer was removed in *vacuo*. The pure product was obtained by silica gel chromatography using EA:MeOH = 10:1 as eluent to afford **D2** (0.599 g) in 47% yield; ^1^H-NMR (DMSO-d_6_), δ (ppm): 1.06 (t, 3H, CH_3_), 3.05 (m, 4H, CH_2_), 3.79 (m, 2H, CH_2_), 7.00 (m, 1H), 7.16 (m, 4H), 7.53 (s, 1H), 7.73 (d, 1H), 7.86 (s, 1H); Elem. Anal. Calcd. for C_20_H_18_N_2_O_2_: C, 75.45%; H, 5.70%; N, 8.80%. Found: C, 75.16%; H, 5.59%; N, 8.60%.

#### 3.3.4. 10-Ethyl-phenothiazine (**8**)

Phenothiazine (**7**: 11.94 g, 60 mmol), iodoethane (10.92 g, 70 mmol), and DMF (80 mL) were added to a 50-mL two-necked glass reactor. The solution was warmed to 75 °C, treated portionwise with potassium *tert*-butoxide (7.86 g, 70 mmol), and then refluxed for 24 h. After water (150 mL) was added, the mixture was extracted with chloroform (75 mL). Crude oils obtained by removing the solvent were purified by column chromatography (silica gels, *n*-hexane/ethyl acetate: 20:1 as eluent) to give **8** as a white solid (11.32 g, yield: 83%). MP: 103–104 °C; ^1^H-NMR (CDCl_3_), δ (ppm): 1.42 (t, 3H, CH_3_), 3.92 (m, 2H, CH_2_), 6.85–6.88 (m, 4H, ar), 7.11–7.16 (m, 4H, ar); Elem. Anal. Calcd. for C_14_H_13_NS: C, 73.97%; H, 5.76%; N, 6.16%; S, 14.11%. Found: C, 73.83%; H, 5.73%; N, 6.13%, S: 14.10%.

#### 3.3.5. 10-Ethyl-3-formylphenothiazine (**9**)

POCl_3_ (14.8 g, 0.097 mol) was added dropwise to freshly distilled *N*,*N*-dimethylformamide (8.6 g, 0.116 mol, molar ratio 1:1.2) at 0 °C in nitrogen atmosphere. A solution of *N*-ethylphenothiazine (**8**, 6.8 g, 0.03 mol) in *N,N*-dimethylformamide (8 mL) was added dropwise to the POCl_3_/DMF complex at 30 °C. The reaction mixture was stirred at 80 °C for 4 h. When the reaction was finished (TLC monitoring), the reaction mixture was cooled to room temperature and poured into ice water. The obtained mixture was neutralized with NaOH until pH = 7–8. The precipitates were separated by filtration and washed with methanol. The crude product was purified by column chromatography (eluent: ethyl acetate/hexane, 1:6). Yield of **9**: 5.36 g (70%); ^1^H-NMR (CDCl_3_), δ (ppm): 1.45 (t, 3H, CH_3_), 3.98 (m, 2H, CH_2_), 6.88–6.98 (m, 3H, ar), 7.09–7.19 (m, 2H, ar), 7.57–7.65 (m, 2H, ar), 9.80 (s, 1H, CHO); Elem. Anal. Calcd. for C_15_H_13_NOS: C, 70.56%; H, 5.13%; N, 5.49%, S, 12.56%. Found: C, 70.49%; H, 5.26%; N, 5.37%, S, 12.38%.

#### 3.3.6. 2-Cyano-3-(10-ethylphenothiazin-3-yl)-acrylic acid (**D3**)

A mixture of **9** (2.01 g, 8 mmol) and cyanoacetic acid (1.36 g, 16 mmol) were vacuum-dried. Acetonitrile (40 mL) and piperidine (0.68 g, 8 mmol) were added to the mixture. The solution was refluxed for 6 h. After the solution was cooled, the organic layer was removed *in vacuo*. The pure product was obtained by silica gel chromatography using EA:MeOH = 10:1 as eluent to afford **D3** (1.31 g) in 51% yield; ^1^H-NMR (DMSO-d_6_), δ (ppm): 1.29 (t, 3H, CH_3_), 3.91 (m, 2H), 6.94–7.23 (m, 5H), 7.65–8.07 (m, 3H); Elem. Anal. Calcd. for C_18_H_14_N_2_O_2_S: C, 67.06%; H, 4.38%; N, 8.69%, S, 9.95%. Found: C, 66.78%; H, 4.30%; N, 8.50%, S, 9.81%.

#### 3.3.7. 3-(10-Ethylphenothiazin-3-yl)-acrylic acid (**D4**)

10-Ethyl-3-formylphenothiazine (**9**, 0.45 g, 1.76 mmol), malonic acid (0.55 g, 5.28 mol), in pyridine (5 mL) were mixed with four drops of piperidine. The reaction was stirred at 90 °C for 2 h, followed by adjustment of pH = 2 with HCl solution. A crude product was obtained and recrystallized from toluene and dried under vacuum. Yield was 91%; ^1^H-NMR (Me_2_SO-*d*_6_), δ (ppm): 1.29 (t, 3H, CH_3_), 3.93 (m, 2H, CH_2_), 6.38 (d, 1H, C=CH), 6.92–7.04 (m, 3H), 7.11–7.22 (m, 2H), 7.44–7.49 (m, 3H); Elem. Anal. Calcd. for C_17_H_15_NO_2_S: C, 68.66%; H, 5.08%; N, 4.71%, S, 10.78%. Found: C, 68.57%; H, 5.18%; N, 4.72%, S, 10.70%.

#### 3.3.8. 2-Cyano-3-(9-ethylcarbazol-3-yl)-acrylic acid (**D1**)

9-Ethylcarbazole (**2**) and 9-ethyl-3-formylcarbazole (**3**) were synthesized with the procedures used for compounds **5** and **6**, respectively. A mixture of 9-ethyl-3-formylcarbazole (**3**, 2.01 g, 9 mmol) and cyanoacetic acid (1.36 g, 16 mmol) were vacuum-dried. Acetonitrile (40 mL) and piperidine (0.68 g, 8 mmol) were added to the mixture. The solution was refluxed for 6 h. After the solution was cooled, the organic layer was removed *in vacuo*. The pure product was obtained by silica gel chromatography using EA/MeOH = 10:1 as eluent to afford **D1** (1.25 g) in 48% yield; ^1^H-NMR (DMSO-d_6_), δ (ppm): 1.33 (t, 3H, CH_3_), 4.49 (m, 2H, CH_2_), 7.27 (t, 1H), 7.52 (t, 1H), 7.66–7.76 (m, 2H), 8.15 (m, 3H), 8.67 (s, 1H); Elem. Anal. Calcd. for C_18_H_14_N_2_O_2_: C, 74.47%; H, 4.86%; N, 9.65%. Found: C, 74.16%; H, 4.76%; N, 9.55%.

### 3.4. Preparation of Dye-sensitized TiO_2_ Electrode (Photoanode) and Counter Electrode

The working TiO_2_ electrode (photoanode) and counter electrode for dye-sensitized solar cells were prepared as follows. F-doped tin oxide (FTO) glass plates (3 mm thickness, 7 Ω cm^−2^) were first cleaned in a cleaning detergent aqueous solution with an ultrasonic bath for 15 min, and rinsed with water and ethanol. Then, the FTO electrodes were immersed into 40 mM TiCl_4_ (aqueous) at 70 °C for 30 min and rinsed with water and ethanol. Two kinds of TiO_2_ paste, containing nanocrystalline (~25 nm) TiO_2_ (Degussa P25, paste A) and submicroparticle TiO_2_ (paste B, 500 nm), respectively, were prepared using a previously reported procedure [40]. To prepare paste A, commercial titania powder (3 g, Degussa P25) was ground in a mortar with a small amount of water (1 mL) containing acetylacetone (0.1 mL), which was added to prevent reaggregation of the particles. After the TiO_2_ particles were dispersed, the paste was diluted by the slow addition of water (3 mL) under continued grinding and a surfactant, Triton X-100 (0.05 mL), was added to facilitate the spreading of the colloid on the substrate. Paste A was sonicated for about 24 h at 28 °C. The spin coating procedure for paste A was repeated to get the appropriate thickness of TiO_2_ films (12 μm). After paste A was dried at 125 °C, paste B was coated two more times, which made the thickness of the TiO_2_ films with 500 nm particles for the scattering layer ~4 μm. The electrodes coated with TiO_2_ pastes were gradually heated (5 °C min^−1^) under airflow up to 450 °C, which was kept for 30 min. The electrodes were treated with 40 mM TiCl_4_. The TiO_2_ films were then rinsed with water and ethanol and sintered again at 450 °C for 30 min. An active area of 0.5 cm × 0.5 cm was selected from a sintered electrode. The electrodes were immersed in a 3 × 10^−4^ M solution of dye containing acetonitrile and isoporpanol (in a volume ratio of 1:1). Dye coatings were applied at room temperature for 24–30 h. The dye-adsorbed TiO_2_ films were taken out and rinsed with dry ethanol. The rinsing process was repeated several times to remove unbound dyes completely. Finally, the dye-adsorbed TiO_2_ films were dried in air. A counter electrode was prepared by sputtering a thin platinum layer on an FTO substrate. The thickness of the Pt layer was controlled to be 50 nm. The thicknesses of the TiO_2_ films were determined by profilometry. Platinum sputtering was carried out using a Hitachi E 1045 instrument. The platinum layer thickness of on the glass substrates was estimated using the amount of sputtered platinum recorded on a quartz thickness monitor.

### 3.5. DSSC Assembly

The dye adsorbed TiO_2_ electrode and Pt-counter electrode were assembled into a sandwich sealed type cell by heating them with hot-melt ionomer film (25 μm thickness, Solaronix) as a spacer. A drop of electrolyte solution [0.1 M LiI, 0.05 M I_2_, 0.6 M DMPII, 0.5 M tert-butyl pyridine (TBP) in acetonitile (ACN)] was injected through a hole in the counter electrode, which was then sealed with hot-melt ionomer film and glass. The electrolyte was introduced into the cell and sealed with AB epoxy for 30 min. The working area of the electrode was 0.25 cm^2^.

### 3.6. Photovoltaic Measurements of the Solar Cells

The photovoltaic measurements of the DSSCs were performed using a Newport M-66907 450 W xenon light source through an infrared blocking filter and a Keithley 2,400 digital source meter linked to a computerized control and data acquisition system. The light intensity was 1000 W·m^−2^ under an AM 1.5 light source. Cell temperatures were kept at 25 °C during the illumination. Light intensity was calibrated using a mono-Si reference solar cell (PVM134). The incident photon-to-current conversion efficiency (IPCE) as a function of excitation wavelength was measured using an incident light 300 W xenon lamp. A 300W xenon arc lamp solar simulator (#91160A, Oriel) with an AM 1.5 Globe filter (#59044, Oriel) was used to measure the I-V characteristics of the quasi-solid-state DSSC. The illumination was fixed at 100 mW·cm^−2^ using a reference solar cell and meter (#91150, Oriel).

### 3.7. Computation Methods

**D1**–**D4** are the carbazole, iminodibenzyl, and phenothiazine-containing dyes, respectively. The geometric and electronic properties of sensitizers **D1**–**D4** were obtained using the Gaussian 03 program package [31]. The calculation was optimized using B3LYP (Becke three parameter hybrid function with Lee–Yang–Perdew gradient correlation functions) with the Pople 6–31 + g(d) atomic basis set. The excitation transitions of **D1**–**D4** were calculated using time-dependent density functional theory (TD-DFT) calculations with B3LYP/6–31 + g(d). Molecular orbitals were visualized using Gaussview.

## 4. Conclusions

Four organic D-π-A dyes by employing various donors (carbazole, iminodibenzyl, and phenothiazine) and various acceptors (cyanoacrylic acid and acrylic acid) were designed and synthesized as the sensitizers for DSSC applications. Significant differences were noticed in the absorption and electrochemical behavior between the cyanoacrylic acid and acrylic acid-based dyes manifest in the DSSCs device performance. Electrochemistry along with DFT calculations revealed that the low-lying LUMO caused by the substitutions is the main reason for the narrowed HOMO-LUMO gaps. The LUMO values of **D1** (−1.70 V), **D2** (−2.14 V), **D3** (−1.92 V), and **D4** (−2.04 V) are more negative than the conduction band edge of TiO_2_ [−0.5 V (*vs.* NHE)]. The HOMO values of **D1** (1.14 V), **D2** (0.77 V), **D3** (0.52 V), and **D4** (0.54 V) are sufficiently more positive than the I_3_^−^/I^−^ redox potential [0.42 V (*vs.* NHE)]. The density functional theory (DFT) calculations reveal that HOMO–LUMO excitation moves the electron density distribution from the donor (carbazole, iminodibenzyl, and phenothiazine) to the acceptor (cyanoacrylic acid and acrylic acid). A solar energy to electricity conversion efficiency of 5.53% is achieved for the phenothiazine and cyanoacrylic acid substituted dye **D3**, which is higher than the corresponding phenothiazine substituted donor-acceptor dye **D4**, and higher than the corresponding carbazole and iminodibenzyl substituted donor-acceptor dye (**D1** and **D2**, respectively). Detailed experiments and the investigation of the interfacial charge transfer processes of these dyes are in progress aiming to further increase the performance of DSSCs fabricated with this new group of dyes.

## Figures and Tables

**Figure 10.**
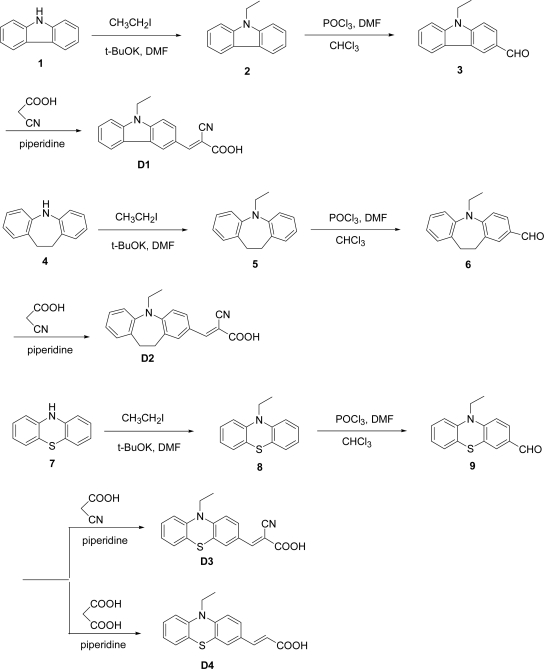
Synthesis of the dyes **D1**–**D4**.

**Table 1.** Absorption and emission properties of dyes.

**Dye**		**Absorption**		**Emission**	**Stokes shift^a^**

	λ_abs_^b^ (nm)	ɛ(M^−1^ cm^−1^) (at λ_abs_)	λ_abs_^b^ (nm) (on TiO_2_)	λ_em_^b^ (nm)	(nm)
**D1**^b^	288, 320, 385	22975 (385 nm)	408	504	119
**D2**	258, 368	29846 (368 nm)	414	470, 506	138
**D3**	299, 400	14096 (400 nm)	426	573	173
**D4**	285, 381	12639 (381 nm)	412	558	177

a Stokes shift = PL_(soluion)_ (nm) – UV_(solution)_ (nm).

b Absorption and emission spectra were measured in CH_3_CN solution.

**Table 2.** Electrochemical properties and band gaps of **D1–D3** dyes.

**Dye**	**E_pa_**	**E_onset(ox)_**	**E_onset(ox)_*vs.* E_FOC_^a^ (V)**	E(S^+^/S*)^b,d^ (V) *vs.* NHE	**E_0–0_^c^ (V)**	**LUMO**	**E_gap_ (V)^e^**
**D1**	1.25	1.11	0.85	1.14	2.84	−1.70	1.2
**D2**	1.00, 1.42	0.74	0.48	0.77	2.91	−2.14	1.64
**D3**	0.72, 1.35	0.49	0.23	0.52	2.44	−1.92	1.42
**D4**	0.63, 1.29, 1.76	0.51	0.25	0.54	2.58	−2.04	1.54

a E_FOC_ = 0.26 V *vs.* Ag/Ag^+^.

b The ground-state oxidation potentials E(S^+^/S) were measured on 0.1 M tetrabutylammonium perchlorate in acetonitrile using a glassy carbon working electrode, a Pt counter electrode, and a Ag/Ag^+^ reference electrode.

c The E_0–0_ value was estimated from the cross-section of absorption and emission spectra.

d The excited-state oxidation potential E(S^+^/S*) was calculated from E(S^+^/S) – E_0–0_.

e E_gap_ is the energy gap between E(S^+^/S*) of the dye and the conduction band level of TiO_2_ (−0.5V *vs.* NHE)

**Table 3.** Comparison of calculated TD-DFT excitation energies (eV, nm), oscillator strengths (*f*), assignment of molecular orbital contributions and character, and experimental absorption band maxima of dyes.

**Dye**	**Excited State**	**f**	**Est (eV)**	**Est (nm)**	**Exp (eV)**	**Exp (nm)**	**Composition**
**D1**	1	0.5501	3.09	400.71	3.22	385	0.17823χ(HOMO-1 -> LUMO) + 0.63923χ(HOMO -> LUMO)
							−0.23908χ(HOMO-2 -> LUMO) −0.10901χ(HOMO-1 -> LUMO)
	2	0.1848	4.05	305.92	3.88	320	
							−0.13026χ(HOMO-1 -> LUMO *+* 2) + 0.60862χ(HOMO -> LUMO *+* 1)
							0.15891 (HOMO-4-> LUMO) + 0.11418χ(HOMO-2 -> LUMO *+* 1)
	3	0.4164	4.62	268.62	4.31	288	
							+0.59488χ(HOMO-1 -> LUMO *+* 1) + 0.21489χ(HOMO -> LUMO *+* 2)
**D2**	1	0.2724	3.80	325.88	3.37	368	0.33984χ(HOMO-3 -> LUMO) + 0.56799χ(HOMO-2 -> LUMO)
	2	0.0519	3.61	343.67			0.69422χ(HOMO-1 -> LUMO)
	3	0.0788	4.69	264.17	4.81	258	0.25285χ(HOMO-5 -> LUMO) + 0.62718χ(HOMO-4-> LUMO)
**D3**	1	0.325	3.46	358.48	3.10	400	0.66021χ(HOMO-1 -> LUMO) + 0.17024χ(HOMO -> LUMO *+* 1)
	2	0.421	4.16	298.32	4.15	299	0.62223χ(HOMO-2 -> LUMO) + 0.23525 (HOMO-> LUMO *+* 3)
							−0.14214χ(HOMO-1 -> LUMO) + 0.64038χ(HOMO -> LUMO + 1)
	3	0.1902	3.85	321.92			
							−0.13726χ(HOMO -> LUMO *+* 2)
**D4**	1	0.3013	2.84	435.84	3.25	381	0.65955χ(HOMO -> LUMO)
							−0.18518χ(HOMO-2 -> LUMO) −0.17372χ(HOMO-1 -> LUMO)
	2	0.3325	4.34	285.39	4.35	285	
							+0.61772χ(HOMO -> LUMO *+* 3)
							−0.10745χ(HOMO-5 -> LUMO) + 0.36767χ(HOMO-4 -> LUMO) +
	3	0.1149	4.60	269.81			
							0.51568χ(HOMO-2 -> LUMO) + 0.12964χ(HOMO -> LUMO *+* 3)

**Table 4.** Photovoltaic performance of DSSCs based on **D1**–**D4** dyes.

Dye	V_oc_ (V)	J_sc_ (mA cm^−2^)	Fill factor (ff)	η (%)
**D1**	0.595	5.76	0.71	2.43
**D2**	0.620	8.28	0.68	3.49
**D3**	0.669	13.35	0.62	5.53
**D4**	0.628	8.09	0.68	3.46

a Measured under irradiation of AM 1.5 G simulated solar light (100 mW cm^−2^) at room temperature, 10 μm film thickness, 0.25 cm^2^ working area.

b The concentration of dye is 2 × 10^−4^ M in CH_3_CN and 0.6 M tetrabutylammonium iodide (TBAI), 0.1 M LiI, 0.05 M I_2_, 0.6 M DMPII, and 0.5 M 4-tert-butylpyridine (TBP) in dry acetonitrile (ACN) as electrolyte.
